# Disruption of the S41 Peptidase Gene in *Mycoplasma mycoides capri* Impacts Proteome Profile, H_2_O_2_ Production, and Sensitivity to Heat Shock

**DOI:** 10.1371/journal.pone.0051345

**Published:** 2012-12-31

**Authors:** Ayman B. Allam, Mary B. Brown, Leticia Reyes

**Affiliations:** Department of Infectious Diseases and Pathology, College of Veterinary Medicine, University of Florida, Gainesville, Florida, United States of America; Leibniz Institute for Natural Products Research and Infection Biology- Hans Knoell Institute, Germany

## Abstract

Members of the *Mycoplasma mycoides* cluster are among the most virulent of the mycoplasmas, causing worldwide economically significant diseases of cattle and goats. A distinguishing phenotype among the members of the cluster is the ability to degrade casein. The MMCAP2_0241 gene, an S41 peptidase, confers the proteolytic phenotype in *Mycoplasma mycoides* subsp. *capri* GM12. In order to determine the impact of disruption of the gene, we used differential proteome profiling to compare the *M. mycoides* subsp. *capri* wild type with a mutant lacking the proteolytic phenotype. Disruption of MMCAP2_0241 resulted in altered phenotypes reminiscent of *M. mycoides* subsp. *mycoides* SC and had significant impacts on the proteome profile of the microbe. The mutant exhibited increased production of hydrogen peroxide, decreased lactate dehydrogenase activity, and increased sensitivity to heat shock.

## Introduction

Members of the *Mycoplasma mycoides* cluster are among the most virulent of the mycoplasmas, causing worldwide economically significant diseases of cattle and goats [1], [2]. Two members of the cluster, *M. mycoides* subsp. *mycoides* Small Colony type and *M. capricolum* subsp. *capripneumoniae* (formerly F38 group), cause contagious bovine and caprine pleuropneumonia, respectively and are listed (http://www.selectagents.gov/select agents and Toxins list.html) by USDA and APHIS (Federal Register 67, No. 155, 9 CFR 121.2b) and the World Organization for Animal Health (http://www.oie.int). Two closely related members of the *M. mycoides* cluster [3], [4], *M. mycoides* subsp. *capri* (formerly *M. mycoides* subsp. *mycoides* Large Colony) and *M. capricolum*, are associated with respiratory disease in small ruminants worldwide and may also cause extrapulmonary complications and sepsis, but these pathogens are not listed agents.

Prior to the advent of genomic analysis, members of the *M. mycoides* cluster were speciated based on key phenotypic differences [5], [6], [7], [8], including colony size on agar, biochemical activities, and thermal stability. A key phenotypic difference between the two species causing contagious pleuropneumonia and other members of the cluster, including *M. mycoides* subsp. *capri* GM12, is the ability to degrade casein [5], [6], [7], an activity that is quite uncommon in mycoplasmas. In addition to the differences in proteolytic activity, *M. mycoides* subsp. *capri* and *M. mycoides* subsp. *mycoides* SC also have different phenotypes with respect to carbohydrate utilization and thermal stability [8]. Further, production of H_2_O_2_ is a key virulence factor in the *M. mycoides* cluster [9], [10], [11], [12], [13], [14], [15], and quantitative differences in H_2_O_2_ production are directly linked to disease severity in mycoplasmas [9], [10], [13].

We identified the gene (MMCAP2_0241) responsible for the proteolytic phenotype in *M. mycoides* subsp. *capri* GM12 ATCC 35297 and created a mutant that has the coding sequence of the gene interrupted by insertion of *tetM* via homologous recombination [16]. *In silico* analysis of the predicted protein product of MMCAP2_0241 indicated a 651 amino acid protein with an unknown N-terminal domain (aa 25–339) and the S41 peptidase tail specific protease (TSP) domain (aa 340 to 544). Within the TSP domain, two carboxyl-tail processing (Ctp) motifs were found from aa 411–422 and aa 477–507. Also present in MMCAP2_0241 was a signal peptide domain (aa 1–24) and two transmembrane domains (aa 7–29 and 631–650). The alignment of MMCAP2_0241 showing conserved domains with other selected CtpA proteins is provided in Fig. S1, supplementary material. Because of the presence of the TSP domain, the presence of Ctp motifs, and homology with similar Ctp proteins in other bacterial species, we previously referred to this gene as *ctpA* [16]. Subsequently, we used the PHYRE (http://www.sbg.bio.ic.ac.uk/phyre2) automatic fold recognition server [17] to evaluate MMCAP2_0241. Based on this analysis, 266 residues (100% confidence, 41% coverage) modeled to the d1fc6a4 template for the superfamily ClpP, which also contains the TSP domain and is a carboxyl-tail processing S41 peptidase [18]. Because *clpP* is known to encode a caseinolytic protease [18], [19], we suggest that *clpP*-like rather than *ctpA* may be the more appropriate designation. However, for consistency with the previous published designation [16], we will refer to the mutant as the *M. mycoides* subsp. *capri ctpA(clpP)::tetM* mutant and to the protein as MMCAP2_0241 (ClpP-like).

Our *M. mycoides* subsp. *capri ctpA(clpP)::tetM* mutant, like the etiologic agents of contagious pleuropneumonia, lacks the proteolytic phenotype [8], [16]. Therefore, it was of interest to determine if disruption of MMCAP2_0241 had significant impacts on the proteome profile of the microbe as well as other phenotypes that differentiate among members of the *M. mycoides* cluster [8]. Here we report that disruption of MMCAP2_0241 resulted in altered phenotypes reminiscent of *M. mycoides* subsp. *mycoides* SC. Specifically, the mutant exhibited increased sensitivity to heat shock as well as increased production of H_2_O_2_. We also observed decreased lactate dehydrogenase (LDH) activity and significant changes in the proteome profile in the *M. mycoides* subsp. *capri ctpA(clpP)::tetM* mutant.

## Results

### Disruption of MMCAP2_0241 resulted in increased sensitivity to heat shock and increased H_2_O_2_ production

As previously reported [16], the *M. mycoides* subsp. *capri ctpA(clpP)::tetM* mutant lost the proteolytic phenotype, as evidenced by lack of enzymatic activity on casein agar; MMCAP2_0241 gene disruption also was demonstrated by both Northern and Southern blots. With respect to carbohydrate use, the *M. mycoides* subsp. *capri ctpA(clpP)::tetM* mutant did not differ from the wild type with respect to fermentation of sorbitol or oxidation of maltose, trehalose, mannose or glucosamine (data not shown).

**Figure 1:**
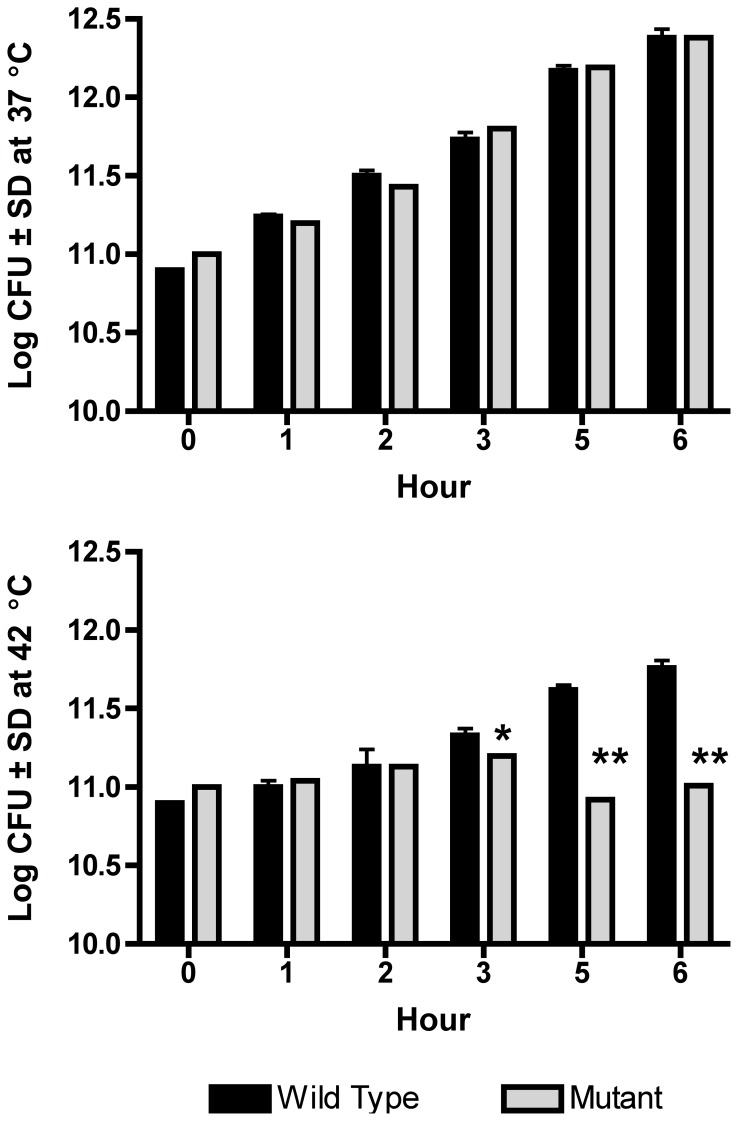
Heat shock response in the *Mycoplasma mycoides capri* GM12 wild type and the *ctpA(clpP)::tetM* mutant. No differences were observed between the mutant and wild type when grown at 37°C. At 42°C, the mutant exhibited significantly reduced growth at 3 hr (*, P<0.01) and subsequent time points (**, P<0.001) than did the wild type.

**Figure 2:**
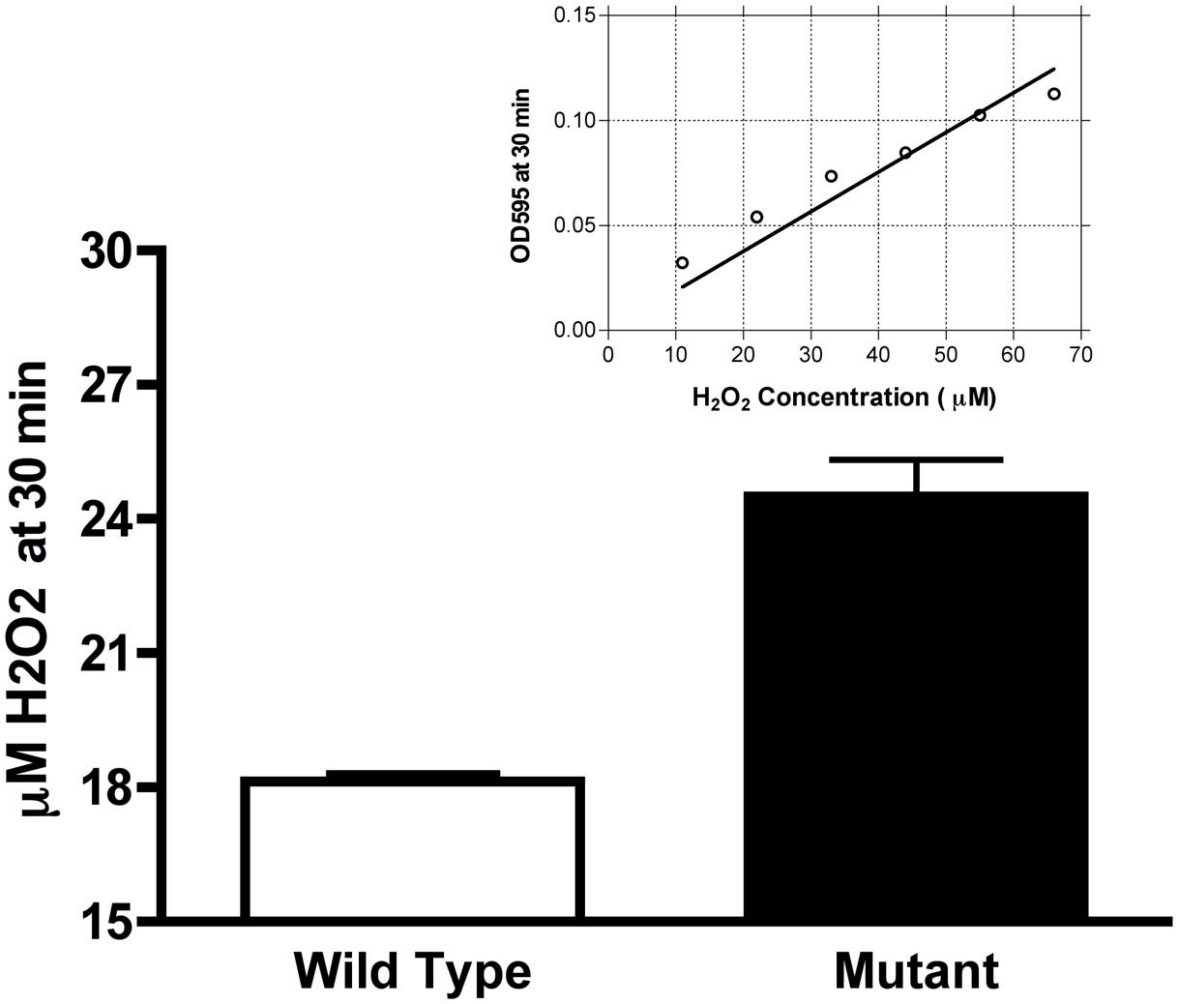
H_2_0_2_ production in the *Mycoplasma mycoides capri* GM12 wild type and the *ctpA(clpP)::tetM* mutant. Production of H_2_0_2_ by 10^8^ cells/ml of either the wild type or mutant was determined after a 30 min incubation with 100 µM glycerol. Production of H_2_0_2_ was significantly increased (P<0.01, unpaired T test) in the mutant. The standard reference curve is shown in the inset.

The disruption of MMCAP2_0241 altered the ability of the mutant to respond to heat stress (Fig. 1), resulting in a phenotype that was more similar to that of *M. mycoides* subsp. *mycoides* SC. At 37°C, the log CFU and growth rates of the wild type and *M. mycoides* subsp. *capri ctpA(clpP)::tetM* mutant were comparable at all time points (Fig. 1). However, after 3 hr at 42°C, the mutant grew more slowly than did the wild type strain (P<0.01). At subsequent time points, the difference was even more dramatic (P<0.001). The CFU of the wild type continued to increase, but the mutant failed to grow, indicating an increased sensitivity to heat shock. The *M. mycoides* subsp. *capri ctpA(clpP)::tetM* mutant exhibited a significant (P<0.01) increase in the production of H_2_O_2_ (Fig. 2), with the mutant producing approximately 40% more H_2_O_2_ than the wild type.

### Disruption of MMCAP2_0241 had a significant impact on the proteome profile

In other microbes, deletion of *ctpA* or *clpP* is associated with pleiotropic effects and proteome differences [20], [21], [22], [23]. Therefore, we next used two complementary approaches, 2D gel electrophoresis/differential gel electrophoresis (DIGE) and amine specific peptide-based labeling (iTRAQ™) followed by tandem mass spectrometry, to identify proteins that were perturbed in the *M. mycoides* subsp. *capri ctpA(clpP)::tetM* mutant. Both methods assess relative differences in the protein concentrations between the *M. mycoides* subsp. *capri* wild type and the *M. mycoides* subsp. *capri ctpA(clpP)::tetM* mutant. However, these two methodologies differ in that DIGE is protein-centric whereas iTRAQ is peptide-centric [24], [25], [26]. There are also discrepancies based on cellular compartmentalization (cytosol vs. membrane), abundance, and limitations inherent to each assay.

In our 2-D gels, eight proteins were found to differ significantly (P<0.006) between the *M. mycoides* subsp. *capri* wild type and *M. mycoides* subsp. *capri ctpA(clpP)::tetM* mutant (Table 1 and Fig. S2, supplementary material). Proteins that were significantly decreased in *M. mycoides* subsp. *capri ctpA(clpP)::tetM* were preprotein translocase SecA, adenylsuccinate synthase, phosphoglycerate kinase, and two conserved hypothetical proteins. The only proteins significantly increased in the mutant were the transcription anti-termination protein NusG and serine-tRNA ligase. TetM was present only in the *M. mycoides* subsp. *capri ctpA(clpP)::tetM* mutant, as expected.

**Table 1:** Proteins that significantly differed (P<0.006) in the *M. mycoides* subsp. *capri ctpA::tetM* mutant as determined by differential 2-dimensional gel electrophoresis.

Spot	Accession	Gene locus	Protein name	Prot. ID prob	Change
1468	GI:256383992	MMCAP2_0840	NusG	99.9%	Increase
1471	GI:256383992	MMCAP2_0840	Nus G	100%	Increase
1472	GI:256383992	MMCAP2_0840	Nus G	100%	Increase
206	GI:256383767	MMCAP2_0095	SecA	100%	Decrease
1844	GI:256384171	MMCAP2_0128	Hypothetical protein	99%	Decrease
602	GI:256384387	MMCAP2_0061	Serine–tRNA ligase	100%	Increase
962	GI:256383734	MMCAP2_0766	Adenylosuccinate synthase	100%	Decrease
1001	GI:256383985	MMCAP2_0606	Phosphoglycerate kinase	100%	Decrease
123	GI:256383953	MMCAP2_0480	Hypothetical protein	100%	Decrease
517	GI:108795342	N/A	TetM [*E. coli*]	100%	Increase

The spot numbers correspond to spot identifications on 2D gels (see Figure S1 in supplementary material). Accession numbers refer to GenBank (http://www.ncbi.nlm.nih.gov/genbank). The protein sequences are available at GenBank (http://www.ncbi.nlm.nih.gov/bioproject?term=PRJNA39245) and via Genomes on Line (http://www.genomesonline.org/cgi-bin/GOLD/index.cgi).

The iTRAQ™ analysis was more revealing than 2D-DIGE analysis in that 221 proteins were identified with 95% confidence and an error factor <2 [27] (for complete dataset, see Tables S1 and S2 in supplementary material). Based on the Protein Pilot™ algorithm [27], 61 proteins were present in significantly changed concentrations (P<0.01) in the *M. mycoides* subsp. *capri ctpA(clpP):tetM* mutant when compared to the wild type (Table 2; Table S2). In order to derive biological meaning from these differences, proteins were grouped according to global biologic functions as assigned in the Molligen 3.0 database [28] (http://cbi.labri.fr/outils/molligen) or UNIPROT [29], [30] (www.uniprot.org/).

**Table 2:** Proteins that were significantly altered by the disruption of MMCAP2_0241 in *Mycoplasma mycoides* subsp. *mycoides* as determined by iTRAQ™.

Gene Locus	Protein Name/Product	Change	Mean Ratio ± SD	Mean P value ± SD
**Environment Information Processing: Membrane transport**				
MMCAP2_0427	Phosphate ABC transporter, ATP-binding protein	Inc	1.357±0.106	0.003±0.004
MMCAP2_0336	PTS system, IIA component	Dec	0.600±0.010	<0.001±1.15E−05
MMCAP2_0360	Signal recognition particle protein (Fifty-four homolog)	Inc	1.487±0.0416	<0.001±4.65E−04
**Genetic Information Processing: Folding, Sorting and Degradation**				
MMCAP2_0065	Thioredoxin	Dec	0.640±0.010	<0.001±7.51E−05
MMCAP2_0213	Phosphopyruvate hydratase	Dec	0.603±0.021	<0.001±5.77E−06
MMCAP2_0545	ATPase family associated with various cellular activities (AAA) protein	Inc	1.373±0.049	<0.001±0.001
**Genetic Information Processing: Replication and Repair**				
MMCAP2_0771	Ribonucleoside diphosphate reductase, alpha subunit	Dec	0.710±0.0264	<0.001±0.001
MMCAP2_0773	Ribonucleotide diphosphate reductase, beta subunit	Dec	0.643±0.021	<0.001±2.00E−05
MMCAP2_0885	tRNA uridine 5-carboxymethylaminomethyl modification enzyme	Inc	1.550±0.095	0.003±0.004
**Genetic Information Processing: Transcription**				
MMCAP2_0407	RNA polymerase sigma-A factor	Inc	1.396±0.038	<0.001±1.50E−04
**Genetic Information Processing: Translation**				
MMCAP2_0025	30S ribosomal protein S18	Inc	1.500±0.100	<0.001±0.001
MMCAP2_0061	Serine-tRNA ligase	Dec	0.630±0.060	0.003±0.002
MMCAP2_0148	30S ribosomal protein S12	Inc	1.390±0.010	0.003±0.002
MMCAP2_0149	30S ribosomal protein S7	Inc	1.337±0.080	<0.001±1.15E−05
MMCAP2_0151	Translation elongation factor Tu	Inc	1.247±0.0321	0±0
MMCAP2_0222	Threonine-tRNA ligase	Dec	0.587±0.021	<0.001±3.00E−05
MMCAP2_0362	30S ribosomal protein S16	Inc	1.427±0.060	0.003±0.001
MMCAP2_0519	Isoleucyl-tRNA synthetase	Dec	0.657±0.040	0.003±0.002
MMCAP2_0644	50S ribosomal protein L17	Inc	1.427±0.021	<0.001±0.001
MMCAP2_0653	50S ribosomal protein L15	Inc	1.457±0.035	<0.001±1.73E−05
MMCAP2_0656	50S ribosomal protein L6	Inc	1.417±0.055	0±0
MMCAP2_0663	50S ribosomal protein L29	Inc	1.470±0.044	0.001±0.001
MMCAP2_0667	30S ribosomal protein S19	Inc	1.397±0.015	0.003±0.002
MMCAP2_0668	50S ribosomal protein L2	Inc	1.303±0.083	<0.001±1.56E−04
MMCAP2_0669	50S ribosomal protein L23	Inc	1.377±0.038	0.001±0.001
MMCAP2_0671	50S ribosomal protein L3	Inc	1.310±0.036	0.001±3.96E−04
MMCAP2_0672	30S ribosomal protein S10	Inc	1.467±0.0764	0.001±0.002
MMCAP2_0806	50S ribosomal protein L7/L12	Inc	1.407±0.040	0±0
MMCAP2_0809	50S ribosomal protein L1	Inc	1.417±0.051	<0.001±3.01E−04
MMCAP2_0810	50S ribosomal protein L11	Inc	1.507±0.027	<0.001±2.25E−04
**Metabolism: Amino acid**				
MMCAP2_0036	D-lactate dehydrogenase	Dec	0.610±0.036	0±0
MMCAP2_0059	Alanine dehydrogenase	Dec	0.680±0.020	<0.001±5.77E−06
MMCAP2_0120	Threonine ammonia lyase	Dec	0.637±0.006	0.001±0.002
MMCAP2_0225	Pyruvate dehydrogenase (acetyl-transferring) E1 component, alpha subunit	Dec	0.807±0.032	<0.001±5.03E−05
MMCAP2_0226	Pyruvate dehydrogenase E1 component, beta subunit	Dec	0.823±0.006	<0.001±0.001
MMCAP2_0519	Isoleucyl-tRNA synthetase	Dec	0.657±0.040	0.003±0.002
MMCAP2_0628	Putrescine carbamoyltransferase	Inc	1.523±0.045	<0.001±1.15E−05
MMCAP2_0765	Adenylosuccinate lyase	Dec	0.560±0.044	0.002±0.002
MMCAP2_0766	Adenylosuccinate synthetase	Dec	0.450±0.036	0±0
MMCAP2_0786	Ornithine carbamoyltransferase	Dec	0.517±0.031	0±0
**Metabolism: Other Amino Acids**				
MMCAP2_0059	Alanine dehydrogenase	Dec	0.680±0.020	<0.001±5.77E−06
**Metabolism: Carbohydrate**				
MMCAP2_0036	D-lactate dehydrogenase	Dec	0.610±0.036	0±0
MMCAP2_0131	Fructose-1,6-bisphosphate aldolase, class II	Dec	0.667±0.064	0.002±0.003
MMCAP2_0213	Phosphopyruvate hydratase	Dec	0.603±0.021	<0.001±5.77E−06
MMCAP2_0225	Pyruvate dehydrogenase (acetyl-transferring) E1 component, alpha subunit	Dec	0.807±0.032	<0.001±5.03E−05
MMCAP2_0226	Pyruvate dehydrogenase E1 component, beta subunit	Dec	0.823±0.006	<0.001±0.001
MMCAP2_0336	PTS system, IIA component	Dec	0.600±0.010	<0.001±1.15E−05
MMCAP2_0451	NADP-dependent glyceraldehyde-3-phosphate dehydrogenase	Dec	0.560±0.010	0±0
MMCAP2_0606	Phosphoglycerate kinase	Dec	0.503±0.006	0±0
MMCAP2_0607	glyceraldehyde-3-phosphate dehydrogenase, type I	Dec	0.797±0.031	0±0
MMCAP2_0733	Phosphoglucomutase or phosphomannomutase	Dec	0.717±0.0389	0.002±0.002
MMCAP2_0831	Ribose phosphate pyrophosphokinase	Dec	0.653±0.0328	<0.001±5.20E−05
**Metabolism: Cofactors and Vitamins**				
MMCAP2_0464	Lipoate-protein ligase A	Dec	0.443±0.015	0±0
**Metabolism: Energy**				
MMCAP2_0344	Inorganic diphosphatase	Dec	0.760±0	0.002±0.002
**Metabolism: Enzyme Families**				
MMCAP2_0188	Oligoendopeptidase F	Dec	0.750±0.023	0.002±0.004
**Metabolism: Nucleotide**				
MMCAP2_0129	CTP synthase	Dec	0.550±0.026	<0.001±1.04E−04
MMCAP2_0765	Adenylosuccinate lyase	Dec	0.560±0.044	0.002±0.002
MMCAP2_0766	Adenylosuccinate synthetase	Dec	0.450±0.036	0±0
MMCAP2_0771	Ribonucleoside diphosphate reductase, alpha subunit	Dec	0.710±0.026	<0.001±0.001
MMCAP2_0773	Ribonucleotide diphosphate reductase, beta subunit	Dec	0.643±0.021	<0.001±2.00E−05
MMCAP2_0831	Ribose phosphate pyrophosphokinase	Dec	0.653±0.032	<0.001±5.20E−05
**Metabolism: Lipid**				
MMCAP2_0218	Glycerol kinase	Dec	0.777±0.015	<0.001±0.001
**Unclassified**				
MMCAP2_0077	Putative hydrolase of the HAD family	Dec	0.720±0.028	0.001±4.01E−04
MMCAP2_0189	Conserved hypothetical protein	Dec	0.685±0.049	<0.001±2.40E−04
MMCAP2_0224	Probable lipoate-protein ligase A	Dec	0.695±0.007	0.001±0.001
MMCAP2_0237	Dihydroxyacetone kinase, phosphotransfer subunit	Inc	2.040±0.056	<0.001±1.15E−05
MMCAP2_0440	Putative liporotein	Dec	0.810±0.014	0.0017±0.002
MMCAP2_0459	Glycerol ABC transporter, glycerol binding protein	Dec	0.455±0.007	0±0
MMCAP2_0460	Conserved hypothetical protein	Dec	0.815±0.007	0.001±0.002
MMCAP2_0480	Conserved hypothetical protein	Dec	0.725±0.035	0.001±0.001
MMCAP2_0699	Peptide methionine sulfoxide reductase	Dec	0.375±0.007	<0.001±3.18E−04
MMCAP2_0700	Putative liporotein	Dec	0.655±0.035	0.001±0.001

The gene locus was identified using the Molligen database and the *M. mycoides* subsp. *capri* GM12, taxon:436113 genome sequence. A protein may be listed in more than one category if it has multiple functions. Accession numbers refer to GenBank (http://www.ncbi.nlm.nih.gov/genbank). The protein sequences are available at GenBank (http://www.ncbi.nlm.nih.gov/bioproject?term=PRJNA39245) and via Genomes on Line (http://www.genomesonline.org/cgi-bin/GOLD/index.cgi). Protein ratios were generated by dividing the spectral intensity of a specific protein in the *M. mycoides* subsp. *capri ctpA (clpP)::tetM* mutant by the spectral intensity of the specific protein in *M. mycoides capri* GM12. Protein ratios were calculated with the Pro Group™ algorithm (Applied Biosystems/MDS SCIEX). Only ratios from the spectra that are distinct to each protein (or protein form) were used for the calculation. Only proteins with 95% or greater confidence and an error factor (EF)<2 were considered. Proteins were decreased in the mutant if the mean ratio was <1.0; proteins were increased in the mutant if the mean ratio was >1.0. Proteins were grouped according to global biologic function as assigned in the Molligen 2.0 database [28] and/or UniProt [29], [30]. Protein ratios were considered significantly different if they had P values <0.01 as determined by the Pro Group™ algorithm [27].

**Figure 3:**
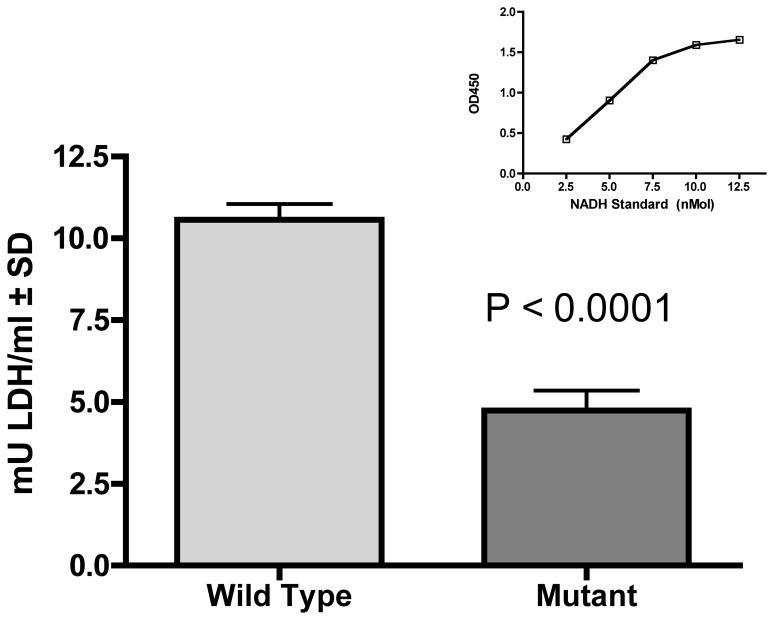
Lactate dehydrogenase production in the *Mycoplasma mycoides* subsp. *capri* GM12 wild type and the *ctpA(clpP)::tetM* mutant. Production of lactate dehydrogenase (LDH) by 10^6^ cells/ml of either the wild type or mutant was determined after a 30 min incubation with 100 µM glycerol. LDH production was significantly increased (P<0.0001, unpaired T test) in the mutant. The standard reference curve is shown in the inset.

An interesting feature in the *M. mycoides* subsp. *capri ctpA(clpP)::tetM* mutant is that most of the significantly altered proteins involved in metabolism were decreased, with the exception of putrescine carbamoyltransferase. Conversely, proteins that were significantly increased in the mutant are associated with genetic and environmental information processing. Proteins with an unassigned unknown function also tended to be decreased. Many of the proteins involved in glycolysis, including LDH, had reduced levels in the mutant. We chose to determine functional activity of LDH because this enzyme is required to convert pyruvate to lactate, thereby recycling NADH. Because mycoplasma lacks the electron respiratory chain, recycling NADH is a key component to maintaining redox potential in the microbe. The *M. mycoides* subsp. *capri ctpA(clpP)::tetM* mutant exhibited about a 50% reduction in LDH activity in comparison to the wild type strain, P<0.0001 (Fig. 3). Thus, the observed functional activity was in agreement with the iTRAQ data for LDH.

## Discussion

Ctps are a group of heterologous serine proteases characterized by resistance to conventional protease inhibitors and a catalytic center that differs from classical serine proteases [31], [32], [33]. Initial studies in bacteria demonstrated that Ctp proteins appear to be multifunctional [34], may be important for degrading damaged or aberrant proteins [35], coping with environmental stress [36], impacting known virulence factors of bacteria [37], [38], [39], [40], [41], and modulating host response to infection [39], [41]. Because Ctp selectively targets and cleaves the non-polar C-terminal of many precursor proteins [40], these enzymes play a role in protein maturation, protein transport into other cellular organelles, or export to the periplasm of Gram negative bacteria [42], [43]. The ClpPs are a S41 peptidase superfamily, also containing the TSP domain. In Gram positive bacteria with low GC content [19], ClpPs are essential in removing heat-damaged proteins. In addition, *clpP* deletion mutants exhibit diverse and pleiotropic effects, ranging from impacts on general stress response, metabolism, sensitivity to high temperatures, metal ion transport, and virulence [19], [20], [22], [23], [44]. In our current study, the disruption of the MMCAP2_0241 gene clearly had diverse, pleiotropic effect, including changes in the proteome profile, decreased proteins involved in metabolism, decreased LDH activity, increased H_2_O_2_ production, and decreased thermal stability. These pleiotropic changes are, for the most part, consistent with the observed effects of other microbial Ctp and ClpP proteins. One notable exception was that in our mutant, production of H_2_O_2_ was increased, whereas in clpP mutants increased sensitivity to H_2_O_2_ was observed [22], [44].

In part because of their limited genome size, mycoplasmas have restricted catabolic pathways, and many of the pathways commonly present in other bacteria are missing or truncated in mycoplasmas [45]. Although glycolysis is the most common central pathway, glycerol also can be used by many mycoplasmas as a carbon and energy source. Glycerol is transported into mycoplasmas, phosphorylated, and then metabolized to dihyroxyacetone phosphate with the concomitant release of H_2_O_2_ [14], [45]. Glycerol oxidation and the subsequent release of H_2_O_2_ plays a central role in the cytotoxicity of *M. mycoides* subsp. *mycoides* SC and the *M. mycoides* cluster [10], [11], [12], [14], [15] as well as in *M. pneumonia* [9], [46]. Increased production of H_2_O_2_ also has been associated with increased virulence and toxicity in other ruminant mycoplasmas, including *M. agalactiae* [13], *M. arginini* [47], *M. bovis* [13], and *M. ovipneumoniae* [47]. European strains of *M. mycoides* subsp. *mycoides* SC are significantly less virulent than African strains and have reduced H_2_O_2_ production [12], [14], [48]. Genomic analysis [14], [48], [49] has shown that the European strains lack an 8.8 Kb DNA segment in the *gtsABC* operon and are less efficient at glycerol uptake, thus explaining the reduced H_2_O_2_ production. A similar *gtsABC* operon was described in *M. leachii* [15], which produces levels of H_2_O_2_ comparable to African strains of *M. mycoides* subsp. *mycoides* SC. However, the production of H_2_O_2_ by itself is not enough to elicit the cytotoxic effect. For example, vaccine strains of *M. mycoides* subsp. *mycoides* SC are able to produce H_2_O_2_ comparable to virulent strains, but have attenuated virulence [10], suggesting that additional virulence factors are required. Based on *in vitro* studies with adhesin-deficient mutants [10], [12], it is likely that strong contact between the mycoplasma and the host cell is required for H_2_O_2_ toxicity, and that this direct contact facilitates the translocation of H_2_O_2_ into the cytoplasm of the host cell. Thus, both adherence to the host cell surface as well as the release of H_2_O_2_ is required for toxicity.

The disruption of MMCAP2_0241 in *M. mycoides* subsp. *capri* impacted a number of proteins, including LDH. The *M. mycoides* subsp. *capri ctpA(clpP)::tetM* mutant had a significant reduction (about 50%) in LDH activity. Although the full biological impact of this reduced activity is not known, it could potentially contribute to oxidative stress. Under normal conditions, *M. mycoides* subsp. *capri* catabolizes sugars to pyruvate via glycolysis [11], [45], [50]. Pyruvate can be reduced to lactate by LDH, which results in reoxidation of NADH to yield NAD^+^ [45]. Because these microbes do not possess an electron transport chain that can be used for this purpose, the recycling of NADH is critical to maintain cellular redox balance [51] and likely to be a crucial point for the adjustment of mycoplasmal metabolism [51], [52].

Another potential source of oxidative stress is reactive oxygen species (ROS) like H_2_O_2_. We observed an approximately 40% relative increase in production of H_2_O_2_ in the mutant. Increased H_2_O_2_ may result in damage not only to the host but also the microbe via oxidation of macromolecules like proteins and lipids. Although most mycoplasmas are deficient in superoxide dismutase and catalase [53], both thioredoxin (TrxA) and methionine sulfoxide reductase (Msr) are present and can modulate oxidative stress and ameliorate the toxic effects of ROS [53], [54]. Interestingly, however, significant decreases as measured by iTRAQ™ were observed in the *M. mycoides* subsp. *capri ctpA(clpP)::tetM* mutant for both Msr (37% decrease) and Trx (65%) proteins, suggesting that the mutant might be under oxidative stress and less able to ameliorate the toxic effects of ROS. The observed increases in H_2_O_2_ levels could be the result of metabolic changes. Most notably, the twofold increase in dihydroxyacetone kinase in the *ctpA(clpP)::tetM* mutant and the decrease in several enzymes in the glycolytic pathway are suggestive of a shift to glycerol metabolism. Because catalase and peroxyredoxin are not present in the genome sequence of *M. mycoides* subsp. *capri*, it is highly unlikely that the increased levels of H_2_O_2_ we observed are due to a decrease in these activities.

The upregulation of ribosomal protein expression under stress conditions has previously been reported in mycoplasmas. The increased expression of ribosomal proteins may reflect the need to increase the translation and protein synthesis. Since the *ctpA(clpP)::tetM* mutant is more susceptible to heat shock and more exposed to oxidative stress, it may experience more protein damage and misfolding. This is consistent with the observed upregulation of ribosomal proteins in response to heat shock in both *M. hyopneumoniae* [55] and *M. pneumoniae* [56]. Additionally, loss of the ctpA/clpP protein could impact the ability of the mutant to degrade damaged or aberrant proteins [35]. Thus, the increased translation could be a compensatory mechanism to cope with these consequences.

Our *M. mycoides* subsp. *capri ctpA(clpP)::tetM* mutant, like the *M. mycoides* subsp. *mycoides* SC, was sensitive to heat shock [8]. Although the mechanism by which MMCAP2_0241 (ClpP-like) is involved in thermal stability in *M. mycoides* subsp. *capri* is unknown, it is interesting that disruption of an *E. coli* gene with a similar TSP-processing domain also resulted in sensitivity to high temperature [57], suggesting that the observed effect might be a result of loss of protein processing.

In our current study, the disruption of the MMCAP2_0241 gene clearly had a pleiotropic effect. Based on our results, it is reasonable to suggest that MMCAP2_0241 (ClpP-like) plays a role in stress response in *M. mycoides* subsp. *capri*. Although the specific mechanisms are not known and may be direct or indirect, it is important to note that by addressing changes in the proteome profile, we were able to identify proteins of interest that would not be predicted based on the loss of the proteolytic phenotype alone. In addition to the loss of the proteolytic phenotype, the mutant also exhibited reduced LDH activity, increased H_2_O_2_ production, and increased susceptibility to heat stress. The increased H_2_O_2_ production is particularly intriguing, as it may have implications for virulence in mycoplasmas [9], [10], [12], [13], [14], [46], [47] and also is a known source of oxidative stress in other bacteria [58].

## Materials and Methods

### Mycoplasma strains and cultivation

*M. mycoides* subsp. *capri* GM12 type ATCC 35297 wild type [1] and the *M. mycoides* subsp. *capri ctpA(clpP)::tetM* mutant that was generated by double cross-over homologous recombination [16] were used in this study. *M. mycoides* subsp. *capri* GM12 type ATCC 35297 has been fully sequenced by The J. Craig Venter Institute, and the full sequences (both gene and protein) are available at GenBank (http://www.ncbi.nlm.nih.gov/bioproject?term=PRJNA39245) and via Genomes on Line (http://www.genomesonline.org/cgi-bin/GOLD/index.cgi). For all experiments, both strains were cultivated in parallel at 37°C in the same batch of SP4 medium, with the exception that the medium was supplemented with 10 µg/ml of tetracycline for growth of the mutant. Microbial growth was monitored by optical density at 640 nm, and cultures were harvested at late log phase (OD_640_ = 0.08). All cultures contained 10^9^ CFU per ml of medium, which was confirmed by direct colony counts. For proteomic and gel electrophoresis studies, cultures were concentrated by centrifugation and used at a final concentration of 10^12^ CFU per ml.

### Preparation of protein extracts for proteomics

Bacterial suspensions were divided into two aliquots: one aliquot was used for 2D gel electrophoresis and the second was used for iTRAQ™ followed by tandem mass spectrometry. Bacterial suspensions were pelleted by centrifugation and washed with wash buffer solution (Calbiochem ProteoExtract® kit, San Diego, CA). For 2D gel electrophoresis, proteins were extracted with Trizol (Invitrogen Corp., Carlsbad, CA) according to the manufacturer's protocol. Pelleted protein extracts were allowed to air dry and were stored at −20°C. For iTRAQ™ analysis, proteins were extracted with ProteoExtract® Complete Mammalian Proteome Extraction Kit (Calbiochem, San Diego, CA) according to the manufacturer's protocol. The total protein concentration of all samples was determined with the Non-Interfering Protein Assay™ Kit (Calbiochem, San Diego, CA).

### 2-Dimensional differential gel electrophoresis (2D-DIGE) and protein identification

Protein pellets were prepared for 2D electrophoresis as previously described [59]. Three 2-D electrophoresis experiments were performed with samples obtained from three independent experiments. The Cy2 internal standard was used to co-detect, match and normalize protein spots in all three gels. Gel images were obtained with Typhoon 9600 Variable Mode Imager (GE Healthcare, Piscataway, NJ) and images were analyzed with DeCyder 2D version 7.0 software (GE Healthcare, Piscataway, NJ). Protein ratios for each gel spot were generated by dividing the total area of *M. mycoides* subsp. *capri ctpA(clpP)::tetM* spot by the total area of the corresponding wild type *M. mycoides* subsp. *capri* spot. Only protein ratios that were 2-fold or greater in difference were considered for further analysis. An automated spot picker (ProPic Workstation, Digilab Genomic Solutions Inc., Ann Arbor, MI) selected protein targets for identification. The same protein spot from each biological replicate was pooled for processing and identification by tandem mass spectrometry as previously described [59]. Tandem mass spectrometric data was searched against NCBI nr bacterial database using Mascot (Matrix Science, Boston, MA) database search engine. Protein identification was performed with Scaffold version 2_01_02 (Proteome Software Inc., Portland, OR). Protein identifications were accepted if they could be established at greater than 80.0% probability and contained at least one identified peptide. Protein probabilities were assigned by the Protein Prophet algorithm [60]. Proteins that contained similar peptides and could not be differentiated based on MS/MS analysis alone were grouped to satisfy the principles of parsimony.

### Quantitative proteomics using peptide labeling and 2D-LC-MS/MS

In order to minimize variability, the protein extracts from all three biological replicates of wild type *M. mycoides* subsp. *capri* (control) were combined and the total protein concentration of the pooled sample was adjusted to match the total protein concentration of each biological replicate of *M. mycoides* subsp. *capri ctpA(clpP)::tetM* mutant. Bacterial protein extracts were prepared as previously described [59] and labeled with an amine specific peptide-based labeling system, iTRAQ™, according to the manufacturer's instructions (Applied Biosystems, Foster City, CA). Labeled samples were analyzed inline with a hybrid quadrupole-TOF mass spectrometer QSTAR (Applied Biosystems Inc) as previously described [59]. Tandem mass spectra were extracted by Analyst (v 1.1.; Applied Biosystem Inc) and the NCBI bacterial protein database (concatenation of the forward and random sequences) was used for protein identification. Searches were performed using MS/MS data interpretation algorithms from Protein Pilot™ (Paragon™ algorithm, v 3.0, Applied Biosystem Inc) and Mascot (v 2.2, Matrix Science, London, UK) as already described [59]. Quantification of protein ratios was based on a minimum of three spectra. Only protein ratios with an error factor (EF) <2 were retained for further analysis. EF is a measure of the variation among the different iTRAQ™ ratios (the greater the variation, the greater the uncertainty) and represents the 95% uncertainty range for a reported ratio. Proteins were grouped according to global biologic function as assigned in the Molligen 2.0 database [28] and/or UniProt [29], [30]. Protein ratios were considered significantly different if they had P values<0.01 as determined by the Pro Group™ algorithm [27].

### Carbohydrate use

We compared the carbohydrate use of *M. mycoides* subsp. *capri* and the *ctpA(clpP)::*tetM mutant using the BBL *Crystal*™ system (Becton Dickinson Microbiology Systems, Cockeysville, Md.) according to the manufacturer's instructions. Both strains were grown to midlogarithmic growth in 2 ml of SP4 broth. Cells were pelleted, suspended in the inoculation medium, the kit test plate inoculated and incubated at 37°C for 24 hr before reading results. Tests were performed in triplicate.

### Response to heat shock

Both wild type and mutant strains were grown to midlogarithmic phase at 37°C as described above. Each culture was divided into two aliquots. One aliquot was kept at 37°C and the other was immediately transferred to 42°C. Each treatment was done in triplicate. At selected time points, an aliquot was removed, serially diluted, and the CFU determined by direct colony count.

### Measurement of H_2_O_2_ production

H_2_O_2_ production was determined as previously described for *M. mycoides* subsp. *mycoides* SC [10]. Six replicates were performed for each strain. Briefly, wild type and mutant *M. mycoides* subsp. *capri* were grown to midlogarithmic phase and harvested by centrifugation at 10,000 rpm for 15 min at 4°C. Bacterial pellets were washed 3 times in incubation buffer (67.7 mM HEPES, pH 7.3; 140 mM NaCl, and 7 mM MgCl_2_). After the final wash, bacteria were resuspended in incubation buffer to a cell density of 10^8^ cells/ml and incubated for 20 min at 37°C. Immediately after the incubation period, glycerol was added to give a final concentration of 100 µM. H_2_O_2_ production was measured at 30 min after glycerol addition using an H_2_O_2_ assay kit (Cayman Chemical, Ann Arbor, MI). The assay, standard curve determination, and specificity quality controls were performed according to the instructional manual.

### Measurement of lactate dehydrogenase activity

Lactate dehydrogenase (LDH) activity and concentration were measured using the Lactate Dehydrogenase Colorimetric Assay Kit (Abcam Inc., Cambridge, MA). Five replicates were performed for each strain. Briefly, 4 ml of both wild type and mutant *M. mycoides* subsp. *capri* were grown to midlogarithmic phase (10^6^ cells/ml) and harvested by centrifugation as described above. The cell pellets were homogenized in 0.5 ml of cold assay buffer, centrifuged at 10,000 rpm for 15 minutes at 4°C, and the supernatant collected. Twenty µl of the supernatant was used in a total 200 µl reaction. A standard curve was constructed, and the LDH activity and concentration were measured and calculated according to the manufacturer instructions.

### Statistical analysis

The 2D gel data, H_2_O_2_ production data, LDH activity, and heat shock growth data were analyzed by unpaired students T test or ANOVA. CFU data was log transformed prior to analysis by ANOVA. For statistical analysis of iTRAQ™ data, protein ratios were generated with Pro Group™ algorithm and automatically corrected for bias. The calculated P-value obtained with the ProGroup™ algorithm is based on 95% confidence interval. A P value<0.05 was accepted as significant.

## Supporting Information

Figure S1 **Alignment and conserved signature sequences of** ***M. mycoides*** **subsp.** ***capri*** **(MMCAP2_0241) and** ***M. capricolum*** **(MCAP_0240) compared with representative bacterial CTP proteins.** *Bacillus subtilis* (ZP_03591706.1), *Bartonella bacilliformis* (YP_988644.1), *Burkholderia pseudomallei* (YP_107067.1), *Escherichia coli* (Prc; M75634.1), *Legionella pneumophila* (YP_122899.1), *Neisseria gonorrheae* (ZP_04720812.1), *Synechococcus* sp. (NP_898059.1), and *Staphylococcus aureus* (BAB42513.1) were used for comparative purposes. Identical and similar amino acid (aa) residues are marked red and blue, respectively. For *M. mycoides capri*, note the signal peptide domain (aa 1–24) followed by the N-terminal domain (aa 25–339), two transmembrane domains (aa 7–29 and aa 631–650) and the S41 peptidase tail specific protease (TSP) domain (aa 340–544). Within the TSP domain and denoted by yellow boxes are the two carboxyl-tail processing (Ctp) motifs (aa 411–422 and aa 477–507). Protein sequences were aligned using Clustal Omega (http://www.clustal.org/omega) followed by analysis using the Sequence Manipulation suite: multiple align show (www.bioinformatics.org/sms). Highly conserved, identical amino acids within the TSP domain are highlighted in red; highly similar amino acids are highlighted in blue. Note that both the amino- and carboxyl- terminal portions of the proteins are quite diverse.
(TIFF)

Figure S2 **Differential 2-dimensional electrophoresis of** ***M. mycoides*** **subsp.** ***capri*** **GM12 wild type and** ***M. mycoides capri ctpA::tetM*** **mutant.** A. The distribution of proteins in the wild type (Cy3 label, red) and mutant (Cy5 label, green). B. The distribution of protein spots that were significantly increased in the mutant. C. The distribution of protein spots that were significantly increased in *M. mycoides capri*.
(TIFF)

Table S1 **Proteins that were not significantly altered by the disruption of** ***ctpA*** **in** ***Mycoplasma mycoides*** **subsp.** ***capri*** **GM12.** The gene locus and gene name, if annotated, were identified using the Molligen database and the *M. mycoides* subsp. *capri* GM12, taxon:436113 genome sequence. GI accession numbers refer to GenBank (http://www.ncbi.nlm.nih.gov/genbank). The protein sequences are available at GenBank (http://www.ncbi.nlm.nih.gov/bioproject?term=PRJNA39245) and via Genomes on Line (http://www.genomesonline.org/cgi-bin/GOLD/index.cgi). Data is shown for three independent biological replicates. Protein ratios were generated by dividing the spectral intensity of a specific protein in the *M. mycoides* subsp. *capri ctpA (clpP)::tetM* mutant by the spectral intensity of the specific protein in *M. mycoides* subsp. *capri* GM12. Protein ratios were calculated with the Pro Group™ algorithm (Applied Biosystems/MDS SCIEX). Only ratios from the spectra that are distinct to each protein (or protein form) were used for the calculation. The total protein score is the measurement of all the peptide evidence for a protein and is analogous to protein scores reported by other protein identification software. Each identified peptide within a protein was assigned a score based on confidence (95% confidence = 2, 95% confidence = 1.3). Raw peptide identification was performed using the Paragon™ database searching algorithm (Applied Biosystems/MDS SCIEX). Raw peptide identification was further processed with the Pro Group Algorithm™ (Applied Biosystems/MDS SCIEX). Only proteins with 95% or greater confidence and an EF factor <2 were considered. Proteins were decreased in the mutant if the ratio was <1.0; proteins were increased in the mutant if the ratio was >1.0. Proteins were considered to be unchanged in the mutant if the P value was >0.001. Further, significance levels had to be obtained in all 3 biological replicates.
(XLSX)

Table S2 **Proteins that were significantly altered by the disruption of** ***ctpA*** **in** ***M. mycoides*** **subsp.** ***capri*** **GM12.** The gene locus and gene name, if annotated, were identified using the Molligen database and the *M. mycoides* subsp. *capri* GM12, taxon:436113 genome sequence. GI accession numbers refer to GenBank (http://www.ncbi.nlm.nih.gov/genbank). The protein sequences are available at GenBank (http://www.ncbi.nlm.nih.gov/bioproject?term=PRJNA39245) and via Genomes on Line (http://www.genomesonline.org/cgi-bin/GOLD/index.cgi). Data is shown for three independent biological replicates. Protein ratios were generated by dividing the spectral intensity of a specific protein in the *M. mycoides* subsp. *capri ctpA (clpP)::tetM* mutant by the spectral intensity of the specific protein in *M. mycoides* subsp. *capri* GM12. Protein ratios were calculated with the Pro Group™ algorithm (Applied Biosystems/MDS SCIEX). Only ratios from the spectra that are distinct to each protein (or protein form) were used for the calculation. The total protein score is the measurement of all the peptide evidence for a protein and is analogous to protein scores reported by other protein identification software. Each identified peptide within a protein was assigned a score based on confidence (95% confidence = 2, 95% confidence = 1.3). Raw peptide identification was performed using the Paragon™ database searching algorithm (Applied Biosystems/MDS SCIEX). Raw peptide identification was further processed with the Pro Group Algorithm™ (Applied Biosystems/MDS SCIEX). Only proteins with 95% or greater confidence and an EF factor <2 were considered. Proteins were decreased in the mutant if the ratio was <1.0; proteins were increased in the mutant if the ratio was >1.0. Proteins were significantly different in the mutant if the P value was <0.001. Further, significance levels had to be obtained in all 3 biological replicates.
(XLSX)
