# Supplementary figures and images for: Disruption of the S41 Peptidase Gene in Mycoplasma mycoides capri Impacts Proteome Profile, H2O2 Production, and Sensitivity to Heat Shock
Source: PLoS One. 2012 Dec 31;7(12):e51345. doi: 10.1371/journal.pone.0051345 (PMC3534093; doi:10.1371/journal.pone.0051345)

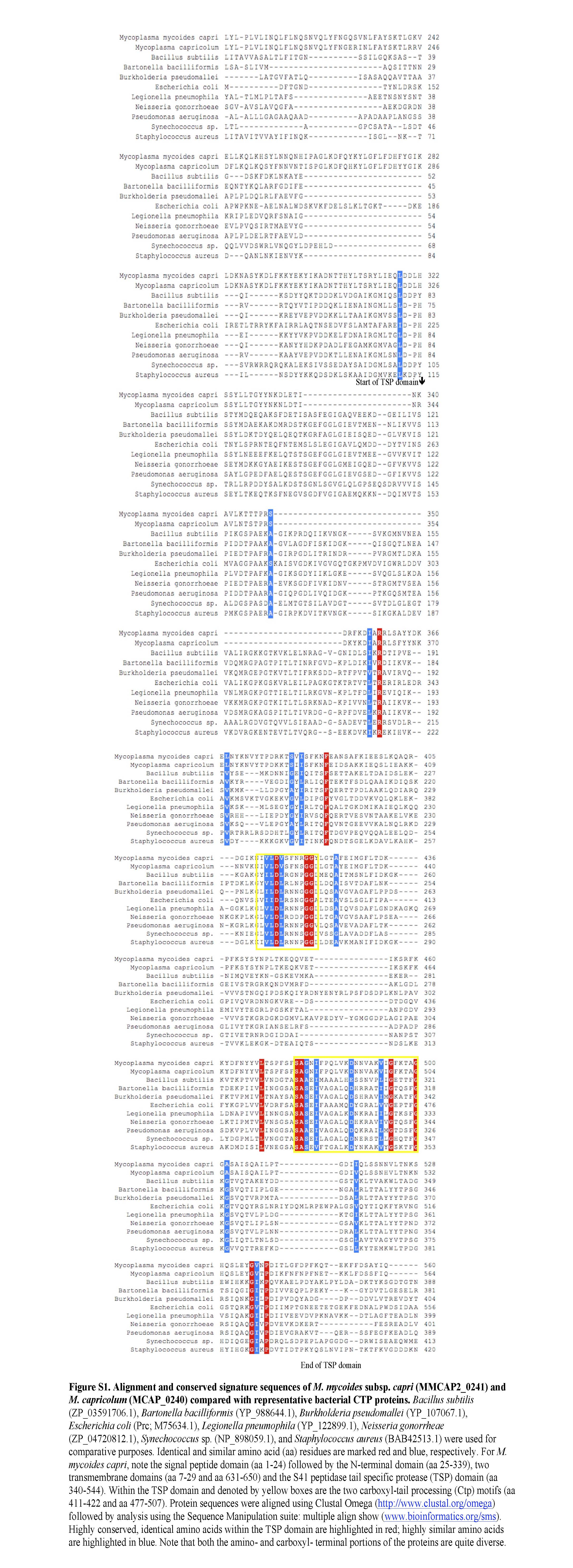

Supplement: Figure S1 — Alignment and conserved signature sequences of M. mycoides subsp. capri (MMCAP2_0241) and M. capricolum (MCAP_0240) compared with representative bacterial CTP proteins. Bacillus subtilis (ZP_03591706.1), Bartonella bacilliformis (YP_988644.1), Burkholderia pseudomallei (YP_107067.1), Escherichia coli (Prc; M75634.1), Legionella pneumophila (YP_122899.1), Neisseria gonorrheae (ZP_04720812.1), Synechococcus sp. (NP_898059.1), and Staphylococcus aureus (BAB42513.1) were used for comparative purposes. Identical and similar amino acid (aa) residues are marked red and blue, respectively. For M. mycoides capri, note the signal peptide domain (aa 1–24) followed by the N-terminal domain (aa 25–339), two transmembrane domains (aa 7–29 and aa 631–650) and the S41 peptidase tail specific protease (TSP) domain (aa 340–544). Within the TSP domain and denoted by yellow boxes are the two carboxyl-tail processing (Ctp) motifs (aa 411–422 and aa 477–507). Protein sequences were aligned using Clustal Omega (http://www.clustal.org/omega) followed by analysis using the Sequence Manipulation suite: multiple align show (www.bioinformatics.org/sms). Highly conserved, identical amino acids within the TSP domain are highlighted in red; highly similar amino acids are highlighted in blue. Note that both the amino- and carboxyl- terminal portions of the proteins are quite diverse. (TIFF) [file pone.0051345.s001.tiff]

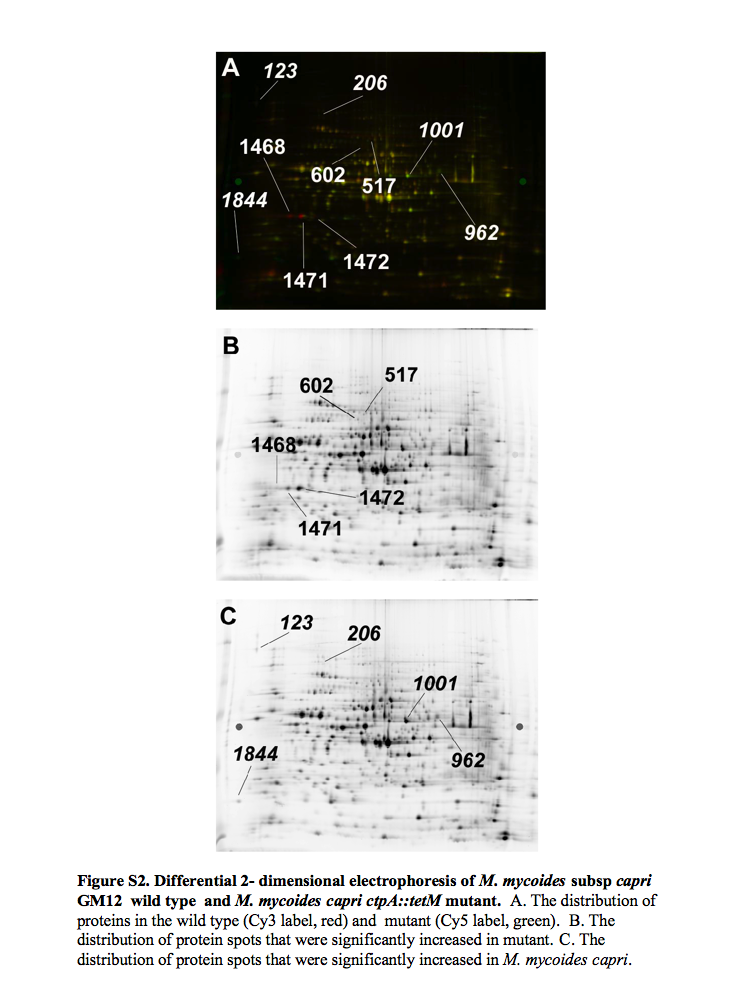

Supplement: Figure S2 — Differential 2-dimensional electrophoresis of M. mycoides subsp. capri GM12 wild type and M. mycoides capri ctpA::tetM mutant. A. The distribution of proteins in the wild type (Cy3 label, red) and mutant (Cy5 label, green). B. The distribution of protein spots that were significantly increased in the mutant. C. The distribution of protein spots that were significantly increased in M. mycoides capri. (TIFF) [file pone.0051345.s002.tiff]
